# Slow Firing Single Units Are Essential for Optimal Decoding of Silent Speech

**DOI:** 10.3389/fnhum.2022.874199

**Published:** 2022-08-03

**Authors:** Ananya Ganesh, Andre J. Cervantes, Philip R. Kennedy

**Affiliations:** ^1^Neural Signals Inc., Neural Prostheses Laboratory, Duluth, GA, United States; ^2^Belize International Institute of Neuroscience, Belize City, Belize

**Keywords:** neural signals, Neurotrophic electrode, single unit firings, speech prosthesis, locked-in participants

## Abstract

The motivation of someone who is locked-in, that is, paralyzed and mute, is to find relief for their loss of function. The data presented in this report is part of an attempt to restore one of those lost functions, namely, speech. An essential feature of the development of a speech prosthesis is optimal decoding of patterns of recorded neural signals during *silent or covert* speech, that is, speaking “inside the head” with output that is inaudible due to the paralysis of the articulators. The aim of this paper is to illustrate the importance of both fast and slow single unit firings recorded from an individual with locked-in syndrome and from an intact participant *speaking silently*. Long duration electrodes were implanted in the motor speech cortex for up to 13 years in the locked-in participant. The data herein provide evidence that slow firing single units are essential for *optimal decoding accuracy*. Additional evidence indicates that slow firing single units can be *conditioned* in the locked-in participant 5 years after implantation, further supporting their role in decoding.

## Introduction

A speech prosthesis is essentially a brain to computer interface between the speech areas of cortex and a computer. More specifically, in the iteration reported herein, it records signals from the motor articulatory area of the brain and transmits these neural signals to a computer that decodes the signals to produce speech in real time. There are several other approaches to developing a speech prosthesis. Most recently, Willett et al. (2021) have recorded from human arm motor cortex and provided a writing facility, easily convertible to speech, using Utah arrays and recording multi-units. Other researchers (Hochberg et al., 2012; Collinger et al., 2013; Kennedy et al., 2017; Jafari et al., 2020; Moses et al., 2021) have used micro-electrode arrays to control paralyzed limbs and robotic limbs, also recording multi-units. In the present report, the Neurotrophic electrode is used to record single units over long time periods (Bouton et al., 2016). Stability of single units has also been reported by Friedenberg et al. (2017).

ECoG electrodes placed on the cortical surface have been used by Chang and his group (Sharma et al., 2015) to develop a speech prosthesis using the frequency domain to interpret and produce the intended speech. In addition, silent/imagined speech was also investigated by Dash et al. (2020a,b) using magnetoencephalography (MEG). In addition, wearable MEG may be a realistic option (Boto et al., 2018). A thorough review of these techniques is outside the scope of this paper and is published elsewhere (Kennedy, 2018).

The locked-in syndrome is consequent on two major conditions: (1) amyotrophic lateral sclerosis is a devastating and slow paralysis; (2) brainstem stroke it is a sudden and complete paralysis. In either case, it leads to loss of use of the articulators, and hence loss of speech, even though the neural substrate in the cortex and sub-cortical areas are intact. These cortical areas are the targets of research efforts to develop a system of stable and long lasting recordings. It follows therefore that only those electrodes that have proven stability and duration should be candidates for human implantation. The discussed systems have been demonstrated to produce functional signals (individual units, multi-units, frequency domain surface recordings) for months and years. Published data on the Utah array, however, indicates loss of 85% of single units after 3 years (Downey et al., 2018) though anecdotal evidence suggests longer duration of multi-unit signals. ECoG array duration was assessed histologically at 22 months with recordings continuing up to 18 months (Degenhart et al., 2016). Histological analysis indicated giant cells and macrophages at the interface, with encapsulation of the electrodes by collagenous tissue. This histology strongly suggests attempts to reject the implant though Pels et al. report no loss of ECoG signal at 3 years (Pels et al., 2019). A more recent configuration is the placement of multiple gold electrodes on the cortical surface, but no long term data has been produced to validate the longevity of this approach (Musk, 2019).

Another approach to avoid rejection of the electrode is to grow the neuropil *inside* the electrode tip. Using this approach, data indicate stability of single units up to 4 years in four participants and 9 years in participant 5 in this study (Kennedy, 2011; Kennedy et al., 2011, 2018). The stability of single unit recordings after 5 years of implantation was such that the participant controlled movements of a cursor in a 2D formant frequency plane to activate vowel sounds (Guenther et al., 2009; Brumberg et al., 2010, 2011), as well as decoding phones and words (Kennedy et al., 2018). Post mortem histological verification 13 years after implantation indicated no gliosis, abundant myelinated axons, and normal neuropil except no neurons (the neurons remain outside the glass tip and grow neurites into the tip under the influence of neurotrophic factors) (Gearin and Kennedy, 2020). The histological analysis result is identical to prior rat and primate studies using light microscopy and electron microscopic techniques (Kennedy et al., 1992). In addition, single units remained functional at year nine (Kennedy et al., 2018). Thus, the question of longevity and stability of recorded single units, as well as the lack of gliosis, has been established with the Neurotrophic electrode.

Advances in decoding with neural network paradigm allow detection of patterns of neural firings. These paradigms allow accurate classification of phones and phonemes described in this paper, and phrases in a prior paper (Kennedy et al., 2018). The classification includes results with slow firing single units as well as fast firing single units. This paper focuses on both fast [5 impulses per second (ips) and above during a 10 s rest period] and slow firing units, with particular emphasis on the importance of slow firing single units for improved accuracy of classification. *There is no human study known to these authors where consistently slow firing single units were specifically related to the task at hand. Some single units are known to cease firing and then burst to a high frequency, but consistently slow, non-bursting units, firing in the 0 to 5 ips range at rest are not known to have been studied, at least with respect to neural prosthesis development*.

In this study, two human participants were implanted with the aim of understanding how to develop a speech prosthesis. The first participant, locked-in due to a brainstem stroke at the age of 16 years, was implanted in 2004. Multiple publications have described the results (Guenther et al., 2009; Brumberg et al., 2010, 2011; Kennedy, 2011; Kennedy et al., 2011, 2018; Gearin and Kennedy, 2020). Even though this locked-in participant (number 5 in FDA IDE G960032) could control his single unit firings, the technology available at that time was not amenable to the production of speech.

To better understand decoding of single units associated with the timing of audible and silent speech, one author (PK) decided to have his speech motor cortex implanted in 2014. An important result from studies in both the locked-in and speaking participants is that even though fast firing units are the most important, slow firing units *optimize* the decoding accuracy. This issue is addressed in this paper. In addition, results from *conditioning of slow* single unit firings illustrate the importance of including slow firing in decoding, so that even if not related to the task they can be conditioned to the task and thus improve decoding capability.

The late David Jayne, an ALS person and friend, insisted that I focus my efforts on restoration of speech, *not movemen*t, because as he said “speech will make me human again. I will talk to my children!”. So the aim of this effort is to restore at least 100 useful words to those who are mute and paralyzed. The expectation is that they speak at a conversational rate, or at least at a rate that allows the locked-in individual to be understood by others during a conversation.

Participant 5 of FDA study (G960032) gave his permission using up movements of his eyes for agreement and down movement for disagreement with the informed consent regulations of Neural Signals Inc. with the regulations being read to him and to his mother and father. His parents also agreed to the regulations. Participant 6 is one of the authors (PK).

## Strategy

Archived data from locked-in participant 5 and intact participant 6 (PK) are analyzed during silent speaking of phones using MATLAB's Classification application (app). This app provides an accuracy rating of the ability to distinguish between individual phones for participant 5 and phonemes plus phones for participant 6. To test the contribution of fast and slow firing single units to the decoding accuracy, fast and slow single units are removed from the analysis one at a time and the accuracy recalculated. In addition, *groups* of fast and slow single units are removed and the accuracy recalculated. For conditioning of slow firing single units, data recorded over several days involved locked-in participant 5 listening to a tone, having a quiet period, and then “singing” it in his head. When feedback was provided, conditioning improved over two sessions as will be shown below, and remained stable.

## Methods

### Electrode

**Figure 1:**
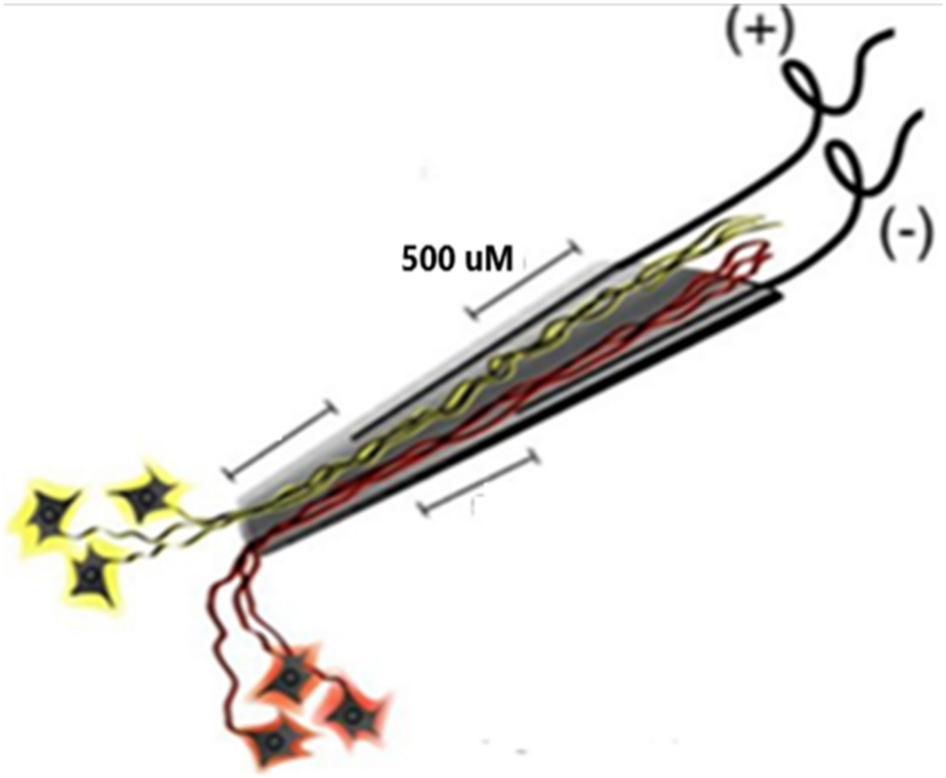
Schematic of the neurotrophic electrode. Ingrowth of neurites is assured by placing trophic factors inside the cone tip prior to implantation. The axons nearest each wire will have opposite polarity during depolarizations.

The electrode assembly is shown in Figure 1. Construction has been detailed (Bartels et al., 2008). The cone is made by pulling a heated pipette to produce tip dimensions of 1.5 mm in length, 25 microns at the deep end and a few hundred microns at the upper end to allow space for the four inserted wires. 2 mil (0.002 inches, 50.4 microns) Teflon insulated gold wires are coiled around a pipette and glued with methyl-methacrylate inside the glass cone. The other end of each coiled gold wire is soldered onto a connector that plugs into the implanted electronic component. The electrodes are FDA approved (IDE G960032).

### Implanted Electronics

**Figure 2:**
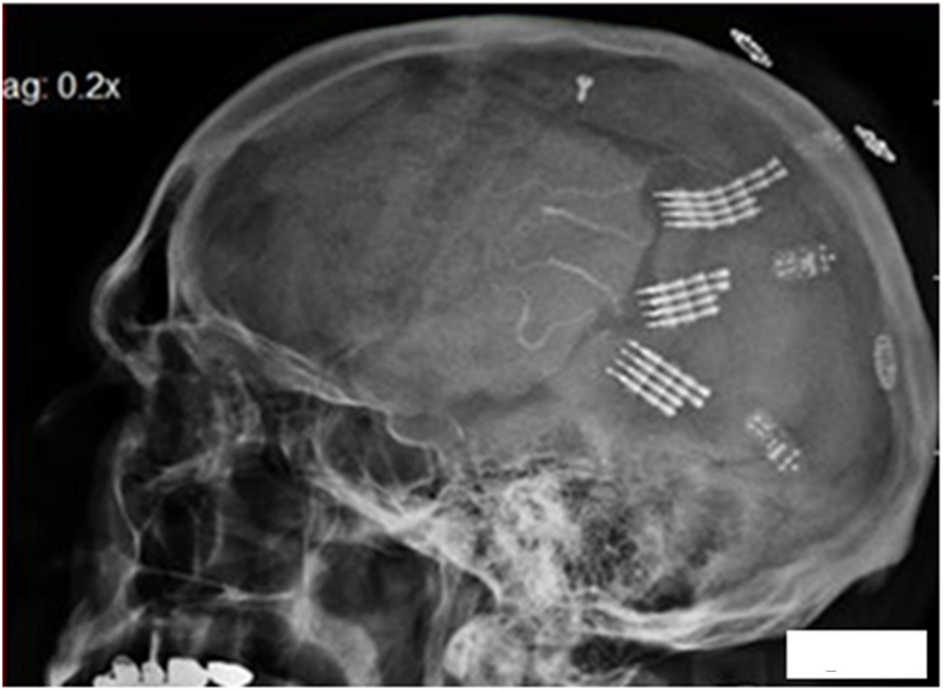
Lateral X ray of the skull in participant 6. The electrode tip are at center, the connecting pins to the right of center, with amplifiers and finally the power induction coils (that appear to be floating). Three pairs of the eight pairs of electrodes wires are attached to three sets of connecting pins that are attached to three electronic amplifiers and FM transmitters.

The single channel electronics are assembled in-house and FDA approved (IDE G960032). Bipolar amplifiers record pairs of wires via the low impedance (50–500 kohms) gold wires that are cut across the tips to provide the low impedances. These connect to an amplifier with a gain of 100 × and the signals are filtered between 5 and 5,000 Hz. The signals then feed into an FM transmitter operating in the carrier range of 35–55 MHz. During recording sessions, a power induction coil powers the device with the induced current passing through a regulator to provide a stable ±3 volts. The electronics are insulated with polymer (Elvax: Ethylene Vinyl Acetate Copolymer Resin, from DuPont, Wilmington Delaware 1998) and further insulated (and protected against trauma during handling while receiving nursing care) with Silastic (Med-6607, Nusil, Silicone Technology, Carpentaria, CA). The gold pin connection to the electrodes is protected with acrylic cement (Medtronic Inc., St. Paul, MN). After implantation, the device is covered with scalp skin. The result is illustrated in Figure 2: a lateral X ray of the skull (in participant 6) indicating three of the eight pairs of electrodes wires are attached to three sets of connecting pins that are, in turn, attached to three single channel electronic amplifiers and FM transmitters. There was insufficient space under the scalp to place more than three electronic devices.

### Implantation Target Site

Because the speech prosthesis is based on movement of the articulators, the speech motor cortex is targeted for implantation. Functional MRI studies during audible and silent speech confirmed the target location as previously described in detail (Kennedy, 2018). This area extends from the Sylvian fissure medially for 30 mm and about 20 mm in the rostro-caudal dimension.

### Surgery

Briefly, under fully sterile conditions, a craniotomy is performed over the left sided speech motor cortex and electrodes are implanted as previously described (Kennedy et al., 2018). The electronics are attached in participant 5 and at a later surgery in the speaking human. Recordings began at month 4 in both participants.

### Recording

The recording systems are detailed elsewhere (Kennedy et al., 2018). Briefly, the power induction coil is placed over the scalp and the underlying receiving coil. It powers the implanted electronics. Data from the FM transmitters are received via a coil placed on the scalp over the transmitting coil. These data are sent to a receiver that sends it on to the Neuralynx (Bozeman, MT) computer that contains the Cheetah cluster cutting system that separates the single units from the continuous data stream. These are then transferred to a laptop computer for off-line analysis employing the Classification App from MATLAB (MathWorks Inc., Natwick, MA).

### Paradigms

**Figure 3:**
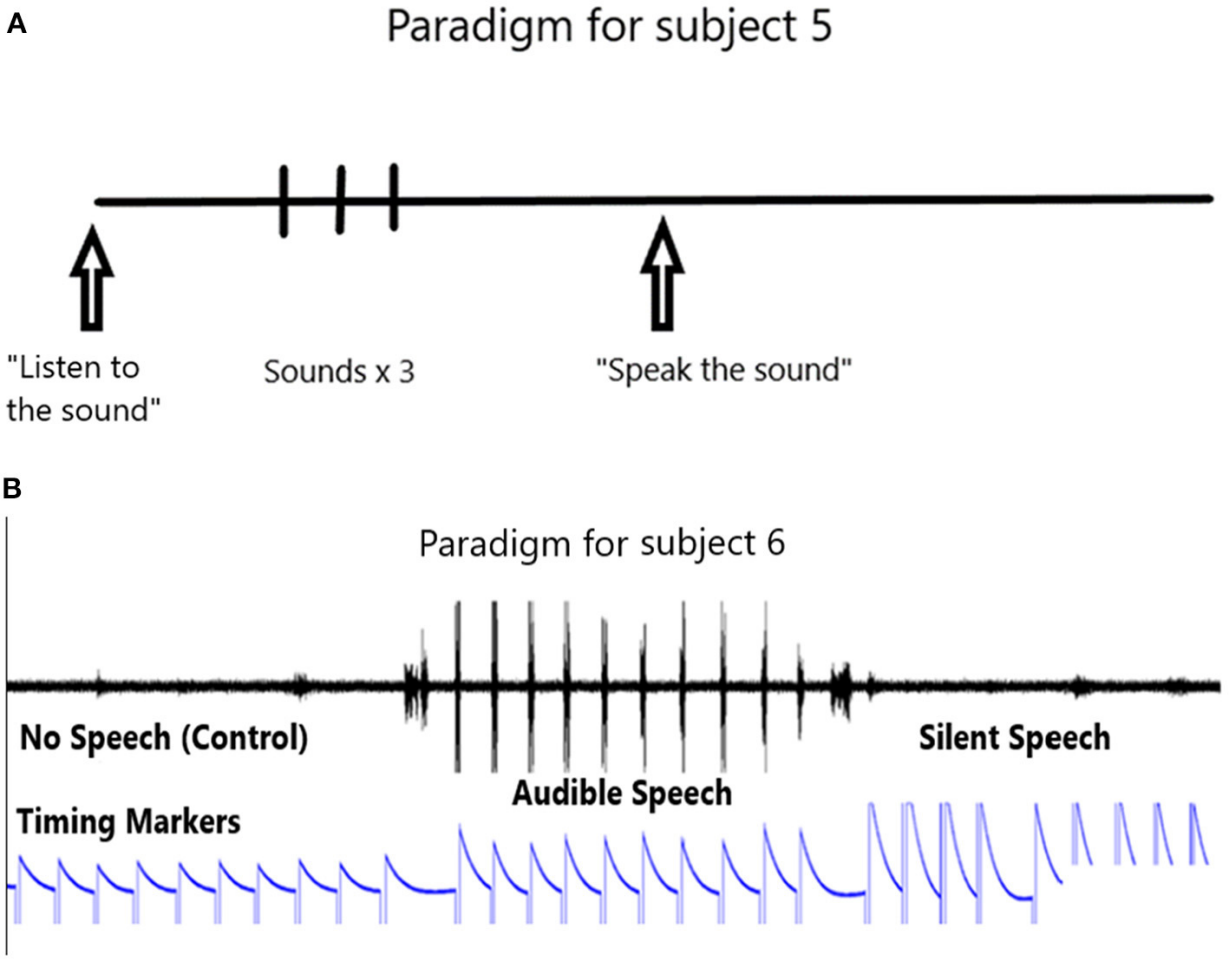
**(A)** Paradigm for participant 5. **(B)** Paradigm for participant 6.

The paradigm used for locked-in participant 5 is shown in Figure 3A. It consists of the computer first outputting an audible phrase “listen to the sound” followed by the phone or word repeated three times. Then the computer emits a phrase “say the sound”. We then assume the participant says the sound in his head. This is repeated ten times. We ask him later to confirm that he did say the phone or word by rolling his eyes up, or deny saying it by rolling his eyes down. The 10 s rest period is taken from the data prior to the active data.

The paradigm used in the speaking human (PK) consists of a control period, followed by a speaking period and then a silent period (speaking a phoneme in the participant's head) as shown in Figure 3B. Note the timing marker that is activated with the left hand. The left hand is preferred to the right so as to minimize possible contamination of the data if the right hand is used (because it is contralateral to the left speech motor cortex). The event markers indicate the control period followed by the audible speech and then the silent speech.

The conditioning study: Participant 5 first listened to a tone for 10 s, and then attempted to sing the tone in his head for 10 s. The rest period is prior to the listening period. Tones are emitted from a sine wave generator as major keys C D G and A in different octaves. All single units are involved in this conditioning study, whereby the participant is asked to sing in his head as accurately as possible. In the first session no feedback is provided. During the second session feedback of one individual single unit firing is provided as a single brief tone each time the single unit fires. During the final session, feedback of single unit firing is provided *and* the volume of the feedback is directly related to the firing rate. Thus, there is a non-linear relationship between the firing rate and the volume of the output.

### Spike Sorting

**Figure 4:**
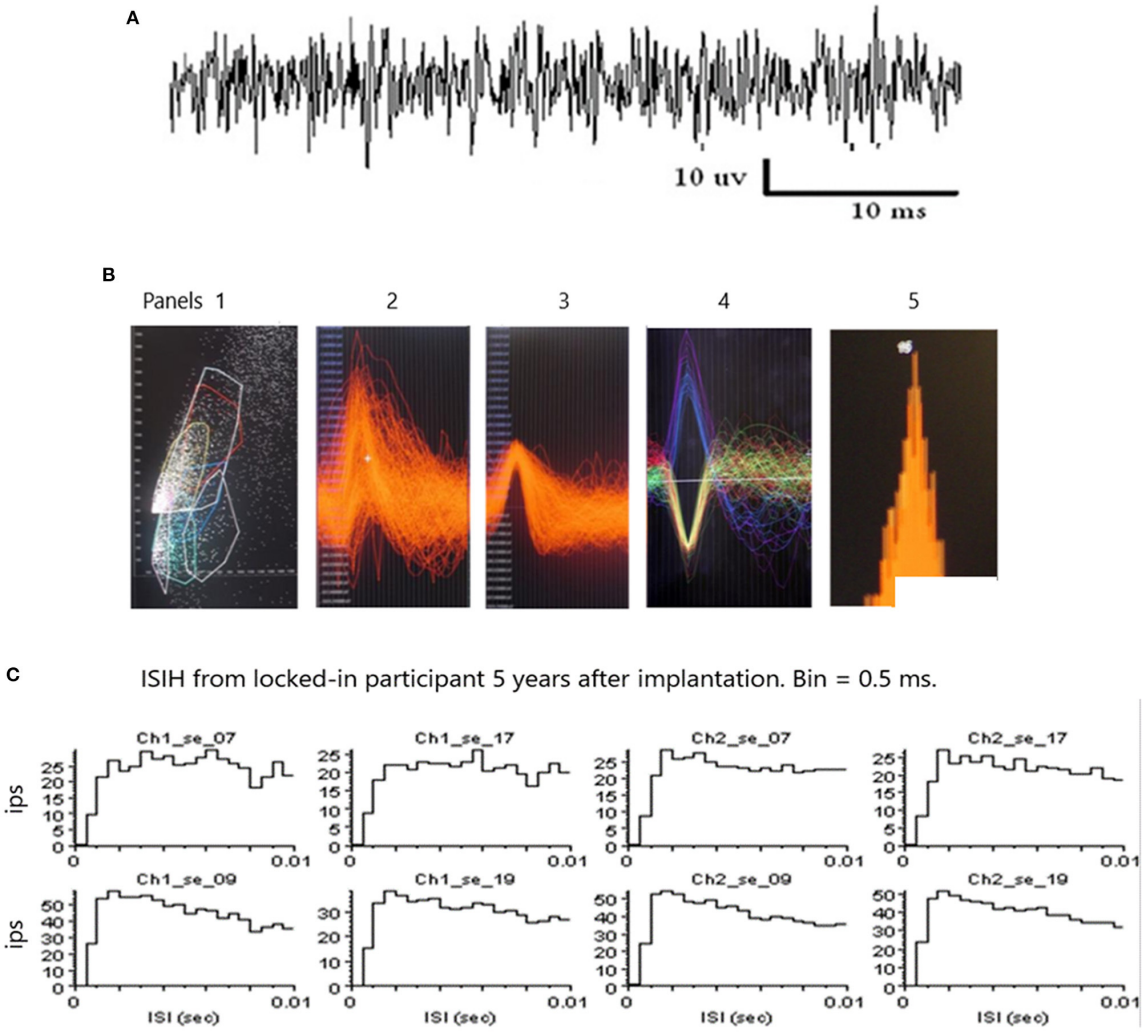
**(A)** An example of continuous data stream from one electrode over a 40 ms timebase. **(B)** Panel 1 illustrates clusters of waveform features (height and width) and a “convex hull” is drawn around a group of dense features. Amplitude is on the abscissa and width on the ordinate. The lower right convex hull surrounds slow firing units. The waveforms emanating from this “convex hull” are displayed in panel 2. Note the white marker that is used to separate the higher amplitude waveforms from the lower amplitude waveforms. Waveforms are separated by placing the marker above and below the peak to remove possible noise, as shown in panel 3. A selection of various waveforms is illustrated in panel 4, some depolarizing in the upward direction and some depolarizing in the lower direction due to the opposite polarity of the recording wires, as shown in Figure 1. The amplitude of the upward depolarizing waveform from baseline is 80 mVs and the time base is one ms. An example of an auto-correlogram of one single unit is shown in panel 5. **(C)** Inter Spike Interval Histograms. Examples of fast firing units indicating the 0.5 ms gap at origin. Slow units will have a false gap and are not shown. Ips, impulses per second. Units taken from both channels of data (ch1_se_07; ch2_se_07). 10 ms time base.

An example of a continuous stream of multi-units neural activity is shown in Figure 4A over a 40 ms time base. Cheetah Spike Sorting software (Neuralynx, Boseman, MT) is employed to sort the continuous data streams into identifiable single units. The preferred algorithm is the convex hull technique which uses a combination of features such as peak, valley, height and area under the curve of the presumptive spikes that are shown in Figure 4B. The program first separates presumed single units into upward or downward action potentials thus creating two channels of data. It then applies the features for single unit separation to each channel (panel 1). The clusters are selected by circling them with the cursor to produce multiple waveforms (panel 2). These are then cut (panel 3) by placing a marker above the presumed waveforms which deletes the outlying waveforms. This technique is further used to remove extraneous signals from the waveform. Finally, examples of various resultant waveforms are shown in panel 4. Time base is 1 ms in panels 2, 3 and 4. These waveforms are then subjected to auto-correlograms to provide further assurance that they can be designated as single units as evidenced by the single peak (one example in panel 5). Inter spike interval histograms are used to verify fast firing units as single units as evidenced by the 0.5 ms gap at origin (Figure 4C). Slow units will have a “false gap”, that is a wider gap due to their random firing and are not shown. Further validation that units are single is dependent on functional studies as described below. Slow firing single units have less dots (panel 1) and are usually larger in amplitude.

### Decoding of Single Unit Patterns

#### Description of the Classification Learner (v.2018b MATLAB)

The Classification Learner is a toolbox containing a variety of classifiers with various algorithms. We use it as follows: When the input vector (set of single units) arrives at the first layer of the network, the distance from the input vector to the training input vectors is computed. This produces a vector whose elements indicate how close the input is to the training input. All classes of inputs are summed to produce as its net output a vector of probabilities. The final step is for the output of the second layer to use a transfer function to produce a 1 for maximum probability and a 0 for the other classes of inputs which thus generates the classification.

#### Application

.txt files containing all the single unit firings in 5, 10, 25 or 50 ms time bins are imported into MATLAB workspace as four different *Tables and analyzed separately*. After decoding passes, 25 ms bins provide the optimal decoding accuracy. The *Classification Learner* app is opened by clicking on the app and then clicking on *New Session* and selecting the imported .txt file (5, 10, 25 or 50 ms). The validation is chosen as 5, 10, 15, 20 or 25 *Fold*. *All* classifier types are selected and the *Train* button is activated. The training is repeated for five validation options (5 10 etc.). Of the many classifiers, the Support Vector Machine (SVM, fine or coarse) and KNN classifiers (k-nearest neighbor) proved to be optimal for decoding these data. Each *.txt* file (corresponding to production of one phone or word) typically contains 500 ms of single unit firings as determined by the acoustic channel that represents audible speech or by the event marker during the control or silent speech periods. One hundred microseconds of data is selected before the acoustic record or event marker because the neural activity has to begin prior to the speech and most likely falls within 100 ms prior to speech onset.

##### Approvals

Approval for the implantation in Belize is obtained from the Belize Committee on Human Experimentation, Belize City, Belize. The participant 5 part of the study is undertaken under IDE G9600032. The study adhered to The Code of Ethics of the World Medical Association (Declaration of Helsinki) for experiments involving humans http://www.wma.net/en/30publications/10policies/b3/index.html; EC Directive 86/609/EEC for animal experiments http://ec.europa.eu/environment/chemicals/lab_animals/legislation_en.htm; Uniform Requirements for manuscripts submitted to Biomedical journals http://www.icmje.org. The Human Investigation Committee of Neural Signals Inc. also approved the study.

## Results

### Phone Classification in the Intact Participant 6 Speaking Silently

Classification is performed as described above using MATLAB.'s app *Classification Learner*. All paradigms [including tree, linear discriminant, quadratic discriminant, support vector machine (SVM), k-nearest neighbor (KNN) and ensemble], are trained by the system. SVM (fine or coarse Gaussian) almost invariably is the most accurate with KNN the next most accurate.

**Figure 5:**
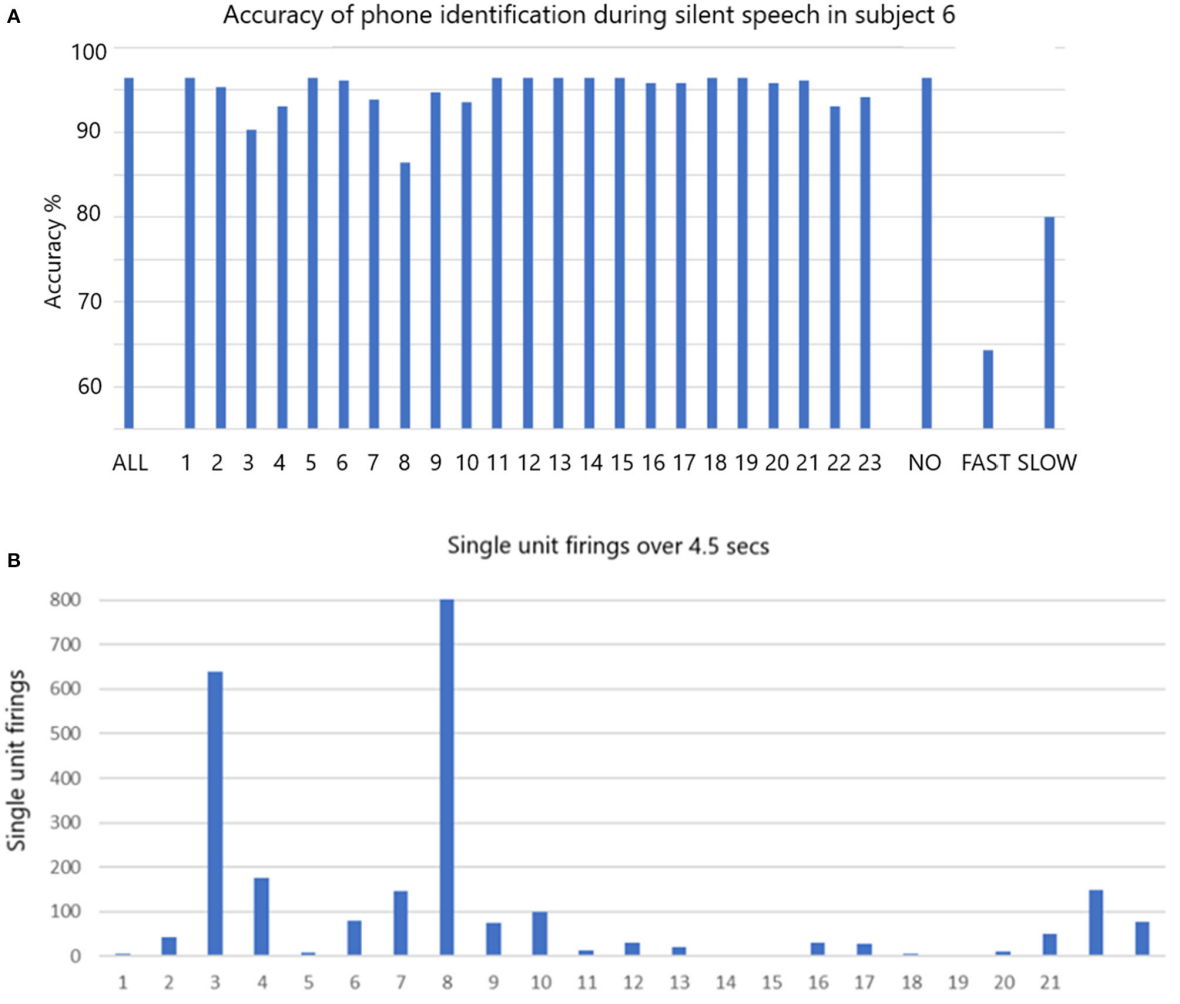
**(A)** The accuracy of phone identification during silent speech in participant 6 is 96.7% when ALL are included (left bar). Excluding each single unit in sequence may change the accuracy. When the non-firing (NO) are excluded the accuracy remains the same. When FAST firing are excluded the accuracy drops as shown. When the slow firing are excluded the accuracy also drops as shown. The fine Gaussian SVM was used for this analysis. **(B)** The total number of firing for all is shown over the whole session that lasted 4.5 s.

The accuracy of phoneme classification is illustrated in Figure 5A for participant 6 *speaking silently*. When all single units are included the accuracy is 96.4% (histogram bar ALL on left). The accuracy varies with removal of each single unit in sequence. When the non-firing single units (non-firing in this task) are removed as a group (#1, 5, 11, 12, 13, 14, 15, 18, 19), the accuracy remains at 96.4% (third histogram bar from right labeled NO). When all the fastest firing are removed (#3, 4, 8, 22, and 23) the accuracy drops dramatically to 64.3% (second bar from right labeled FAST). When slow firing are removed as a group (#2, 6, 7, 9, 10, 16, 17, 20, and 21 labeled SLOW) the accuracy drops to 80% (final bar on right). Data indicating the total firings for each of the 23 single units over 4.5 s are illustrated in Figure 5B. The data indicates that the fast firing single units are more important than the slow firing units, but accuracy is impacted when the slow firing units are excluded.

### Phoneme Classification in Locked-In Participant 5 Speaking Silently

**Figure 6:**
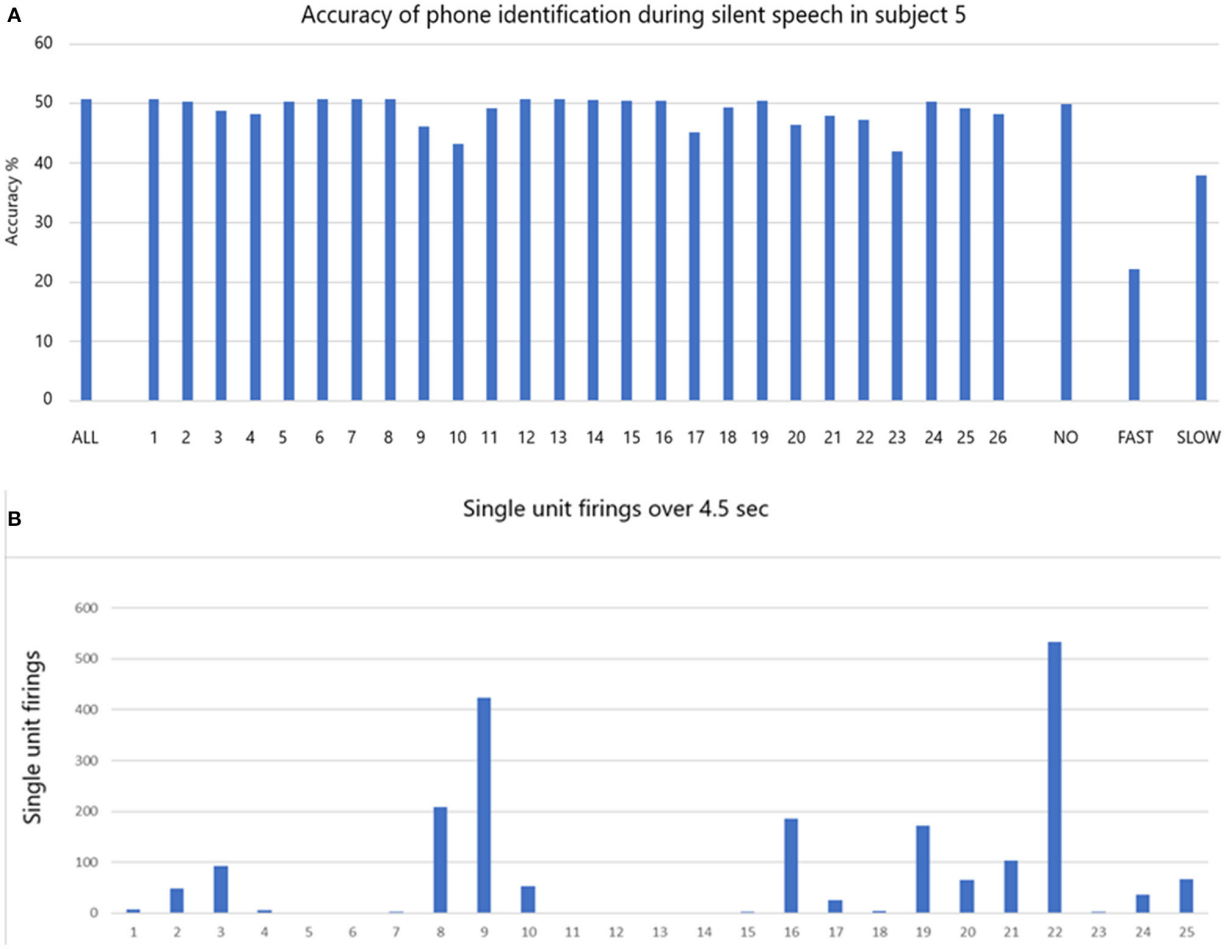
**(A)** The accuracy of phone identification during silent speech in participant 5 is 50.7% when ALL are included (left bar). Excluding each single unit in sequence may change the accuracy. When the non-firing single units are excluded (NO) the accuracy remains the same. When FAST firing are excluded the accuracy drops as shown. When the slow firing are excluded the accuracy also drops as shown. **(B)** The total number of firing for all is shown over the whole session that lasted 4.5 s.

The accuracy of phoneme classification is illustrated in Figure 6A for participant 5 during silent speech. All 39 phonemes were used in the analysis. When all single units are included the accuracy is 50.7% (ALL). The accuracy varies with removal of each single unit in sequence. When all the non-firing (in this task) single units (1, 6, 7, 8, 12, 13, 14, 15, 16, 19, 24) are removed the accuracy is virtually unchanged at 49.9% (NO). When all the fastest firing (9, 10, 17, 20, 21, 22, and 23) are removed the accuracy drops dramatically to 22.2% (FAST). When slow firing are removed as a group (2, 3, 4, 5, 11, 18, 25, 26) the accuracy drops to 37.9% (SLOW). Data indicating the total firings for each of the 26 single units over 4.5 s are illustrated in Figure 6B.

### Conditioning of Slow Firing Single Units

**Figure 7:**
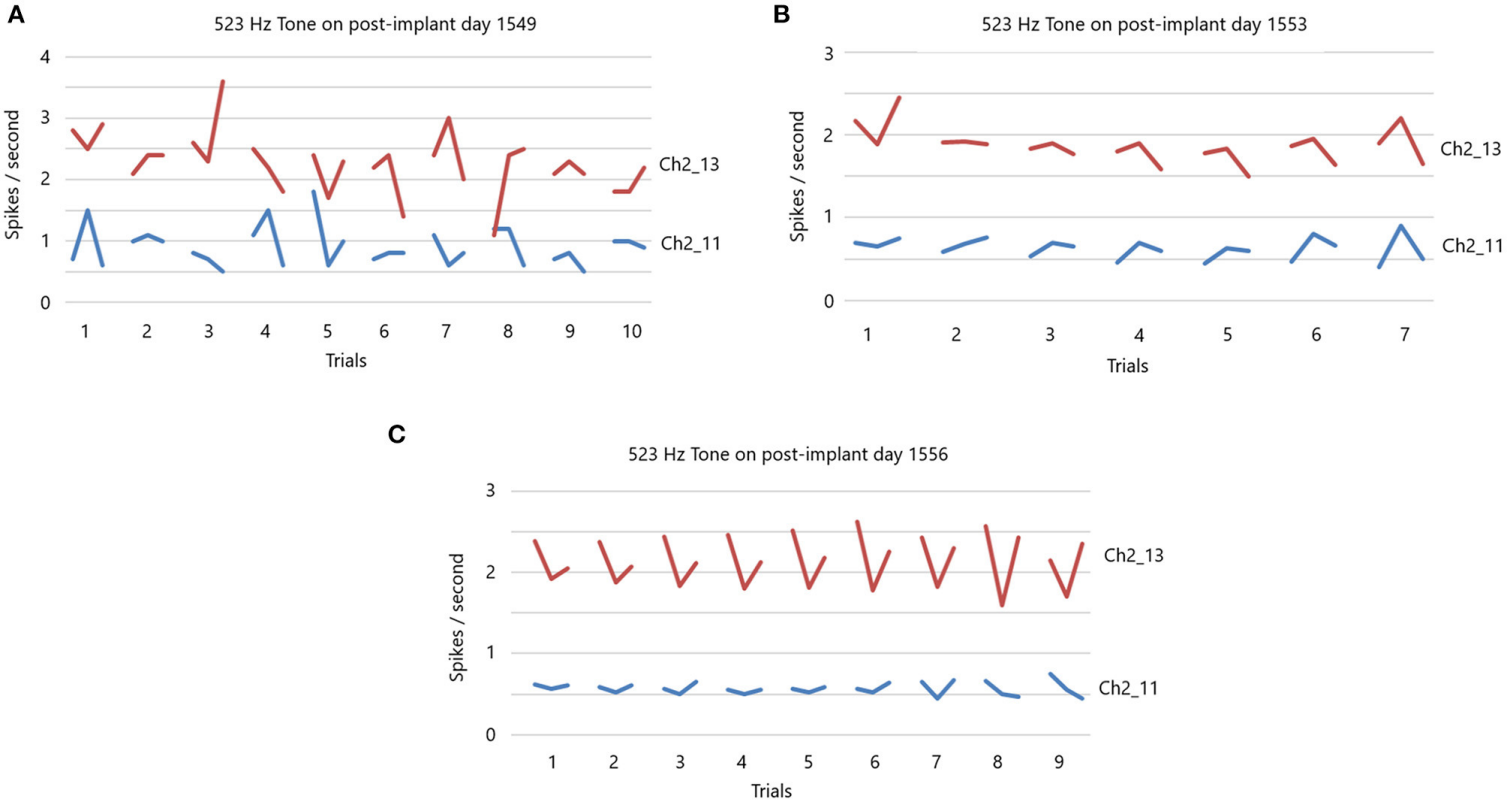
**(A)** 523 Hz tone is presented on post-implant day 1,549. There are 10 trials, with each trial having a 10 s “listen” period, followed by a 10 s rest period and then a 10 s “sing the tone he just heard” period. Note the random firing rates of these slow participant. There is no feedback during this session. **(B)** 523 Hz tone is presented on post-implant day 1,553. Same paradigm as in **(A)** for seven trials. There is feedback of the firing rate. Note the less random firing rates. **(C)** 523 Hz tone is presented on post-implant day 1,556. Same paradigm as in **(A)** for nine trials. There is feedback of the firing rates that is dependent on the feedback volume of the firing rates. Note the non-random firing rates.

In light of the above results, it should follow that the slow firing single units have some functional value. If they have value then it should be possible to condition them. In a different set of studies in locked-in participant 5 four years after implantation, conditioning of slow firing single units is achieved (Kennedy et al., 2011). This study involves silently singing a tone as described in Section Methods. The data in Figures 7A–C are extracted from the data in a prior study (Kennedy et al., 2011). We focus here on the slow firing single units only. Illustrated is the result of presenting a 523 Hz frequency tone (high C).

**Figure 8:**
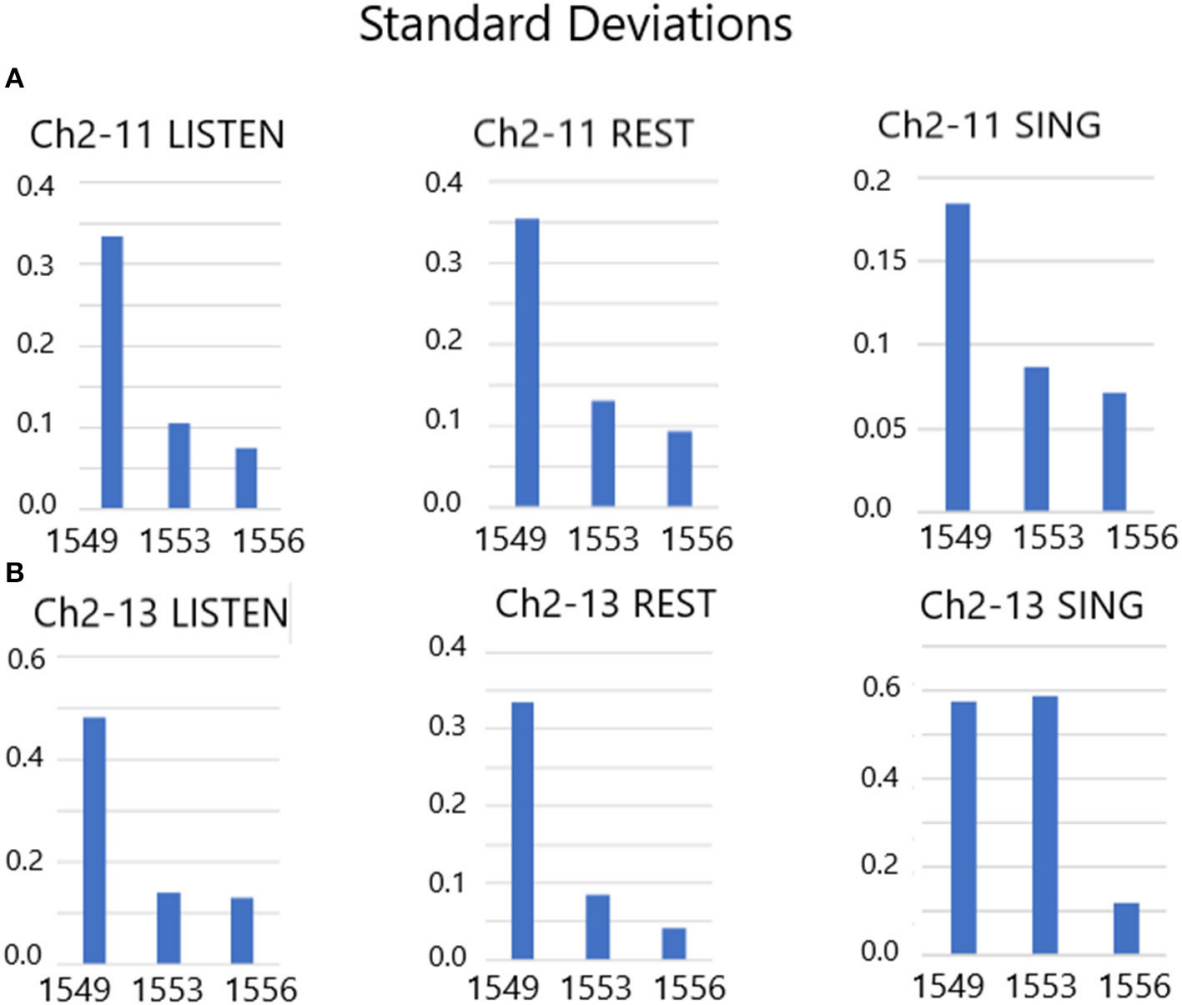
**(A,B)** Standard deviations of Listen, Rest and Sing average firing rates from days 1,549, 1,553 and 1,556. These results indicate a drop in the standard deviation of firing rates during audible feedback conditioning compared with no feedback.

There are 10 separate trials in Figure 7A. The firing rate in spikes per second are shown for the listen period, the rest period and then the sing period (10 s each), followed by a 30 s pause until the next presentation. Clearly, the firing rates (without feedback) of these two slow firing units are random on day 1,549 after implantation. The next session, on day 1,553, audible feedback of the firing rate is presented to the participant. As Figure 7B illustrates, progressing through the session *and* compared to the previous session on day 1,549, the firing rates became much less random. On day 1,556, the audible feedback of the firing rate increased in volume as a function of *increasing* firing rate. Clearly each trial produces a symmetry of the firing rates (Figure 7C). Note that the firing rates do not exceed 4 ips. The standard deviations of the firing rates are plotted in Figure 8 for listen, rest and sing periods for days 1,549, 1,553 and 1,556. There is a clear decrease in standard deviations during the introduction of feedback. Also noted is that the “rest” firing rates are the same on days 1,553 and 1,556, but the firing rates are higher during listen and sing phases for day 1,556 when volume feedback was active.

## Discussion

The data presented above indicate that decoding accuracy is optimal when all single units, slow as well as fast, are included in the analysis. Dropping out individual single units, or groups of single units, reduced the accuracy as shown in Figures 5A, 6A. This was more marked for fast firing single units, but also very noticeable for slow firing single units. The importance of this finding is highlighted when considering the clear drops in accurate decoding.

This finding applies to decoding single units: It was not tested for decoding in the frequency domain.

The difference in the results between participants 5 and 6 is not fully understood. Likely it is due to the fact that participant 5 was locked-in whereas participant 6 was not. We know by direct observation that participant 6 was implanted in the motor speech cortex (30 × 12 mm lateral most part of the left primary motor cortex) whereas participant 5 was not implanted in that area, but was implanted in the general speech area perhaps in the premotor area. Thus, participant 5's signals would not all be related to *motor* speech.

Conditioning of single units implies that single units can be used for functions that are not their original function. Conditioning of fast and slow single units has been achieved (Kennedy et al., 2018). To buttress the present result, an example is extracted from the 2011 paper to illustrate this point in Figures 7A–C for slow firing single units only. These data illustrate that using auditory feedback, the locked-in participant is able to condition the single unit firings as illustrated in Figure 7B and more impressively in Figure 7C. It is worth noting that the feedback involved only the audible firing rate and the participant had no visual representation of the result. In fact, neither the researchers nor the participant had any idea that the participant was conditioning the single units while these data were being acquired. We only asked him to sing the tone in his head on a “look see” basis. It was not until the analysis was completed offline that we understood that the single units were being conditioned. The importance of conditioning of slow or fast firing single units is that they can be conditioned to a new task such as vocalization, movement or other tasks. It follows that just a few (5–10) single units can be used to decode the phone, word or phrase, as demonstrated by the data above. This implies that even though thousands of units can be used for decoding (Hochberg et al., 2012; Collinger et al., 2013; Jafari et al., 2020; Willett et al., 2021), a large number of units are NOT needed for *adequate* decoding, though the accuracy improves with involvement of a larger number of units. Clearly, more units improves the resolution, but are not essential for *adequate* decoding as described here and elsewhere (Brumberg et al., 2010).

Slow firing single units are infrequently analyzed in brain computer interfacing research. One reason is that when trying to develop a motor prosthesis, there needs to a driving force to drive velocity, for example, emanating from the neuronal activity and only fast firing units can provide a high velocity related driving force. Slow firing units could only supply a low velocity related driving force that would be of limited usage. For that reason slow firing single units are rarely described. However, they have been described in the hippocampus in relationship to theta rhythmic clock-like activity (Zhang et al., 2016). The present data strongly suggests they have a role in the *accurate decoding of patterns* of neural activity in a *speech* prosthesis. Theoretically, they may also be useful in precise digit movement generation.

### Alternative Approaches

Undoubtedly, the Blackrock (Utah) microarray and other tine type electrodes have produced remarkably useful single unit firings over months and years (Hochberg et al., 2012). However, 85% of the single units are lost after 3 years (Downey et al., 2018). Thus, the Utah array and similar electrodes are not presently suitable as *long term* recording electrodes, with long term defined as a minimum 50 years. Thus, they would not be suitable for restoration of speech over the long term.

Many other workers in the field take a different approach to decoding neural activity. The ECoG approach of various workers (Conant et al., 2014; Sharma et al., 2015; Herff et al., 2020; Makin et al., 2020) involves decoding spoken words and phrases using cortical surface electrical activity, clearly a data set with less resolution compared to single units. Their data indicate the most effective recording resulted from electrodes over the ventral motor speech cortex (Conant et al., 2014), which is the cortical area targeted for implantation in both the present participants. Conant et al. (2014) did not show that silent speech, (defined again as speaking “in the head” but not using lips, tongue and jaw, and it is not imagined speech) could be detected because they used movements of the articulators without producing sound (they called it miming) which is obviously not realistic for paralyzed and mute participants. However, a more recent study demonstrates that phrases could be decoded using ECoG electrodes over the primary motor and sensory cortex in a locked-in participant (Sharma et al., 2015). So ECoG recording can be used to decode speech in locked-in participants. The main problem with ECoG, however, is that the ECoG electrodes are not compatible with the cortical surface that always rejects foreign bodies. As described in the introduction, however, Pels at al report continuing operation of ECoG at 3 years (Pels et al., 2019).

Other researchers (Balaji et al., 2017; Lawhern et al., 2018) are using EEG recordings allied with deep learning paradigms. It is unlikely that artificial intelligence (AI) will adequately interpret the EEG signals to produce free flowing speech because the spatial resolution with external EEG recordings is too low. Whether or not AI can compensate for this low spatial resolution remains to be seen, but it is unlikely to fully compensate, as recently reviewed (Kennedy, 2018). Other techniques include near field infra-red (NFIR) that penetrates through the skull and measures the blood flow within the cortex as used by Chaudhary et al. (Chaudhary et al., 2017). Again, low resolution is the issue for this technique: useful information is unlikely to be decoded for speech. Other recent developments include tightly spaced multi-electrodes from Neuralink (Musk, 2019). These are likely to suffer gliosis similar to all other penetrating electrodes with subsequent loss of useful signal (Downey et al., 2018). The same gliosis issue will likely be true for the stereotaxic-EEG electrodes (Herff et al., 2020). Other approaches include “neural lace” whereby micro devices powered by ultrasound record directly from neural tissue (Neely et al., 2018). Longevity has not been published but gliosis may intercede between the device and the neural surface. Efforts to minimize the mechanical rigidity of the electrode and thus minimize rejection are underway (Zhao et al., 2019). In summary, these data indicate that for human prosthesis *lifetime* usage there is as yet *no alternative* to the Neurotrophic electrode.

## Conclusion

Slow firing single units are just as important as fast firing units for *optimal* decoding accuracy. The fast firing units when removed from the analysis result in a greater degradation of the accuracy compared with the slow firing single units. The ability to condition single units implies that unrelated single units could be made to relate to the decoding task thus enhancing the bandwidth of the brain to computer interface.

## Data Availability Statement

The raw data supporting the conclusions of this article will be made available by the authors, without undue reservation.

## Ethics Statement

The studies involving human participants were reviewed and approved by Neural Signals Inc. IACUC Committee. Chairman is Prof. T. R. Nichols at Georgia Tech, Atlanta, GA. The patients/participants provided their written informed consent to participate in this study.

## Author Contributions

AG analyzed the data. AC implanted the electrodes in the participants. PK built the electrodes, performed data collection and analysis, and wrote the paper. All authors contributed to the article and approved the submitted version.

## Funding

The authors declare that this study received funding from Neural Signals Inc. The funder was not involved in the study design, collection, analysis, interpretation of data, the writing of this article or the decision to submit it for publication.

## Conflict of Interest

PK has 98% ownership of Neural Signals Inc. AG is employed by Neural Signals Inc. The remaining author declares that the research was conducted in the absence of any commercial or financial relationships that could be construed as a potential conflict of interest.

## Publisher's Note

All claims expressed in this article are solely those of the authors and do not necessarily represent those of their affiliated organizations, or those of the publisher, the editors and the reviewers. Any product that may be evaluated in this article, or claim that may be made by its manufacturer, is not guaranteed or endorsed by the publisher.
