# Supplementary material for: Slow Firing Single Units Are Essential for Optimal Decoding of Silent Speech
Source: Front Hum Neurosci. 2022 Aug 3;16:874199. doi: 10.3389/fnhum.2022.874199 (PMC9382878; doi:10.3389/fnhum.2022.874199)
Supplement: Supplementary file 1 [file Data_Sheet_1.docx]

APPENDIX

Lists of phones

Consonant sounds:

/b/

/d/

/f/

/g/

/h/

/j/

/k/

/l/

/m/

/n/

/p/

/r/

/s/

/t/

/v/

/w/

/y/

/z/

Consonant Digraphs:

/th/ (not voiced)

/th/ (voiced)

/ng/

/sh/

/ch/

/zh/

/wh/

Short vowel sounds:

/a/

/e/

/i/

/o/

/u/

Long vowel sounds:

/a~/

/e~/

/i~/

/o~/

/u~/

Other vowel sounds:

/oo/

/o~o~/

Vowel Diphtonngs:

/ow/

/oy/

Vowel sounds influenced by ’r’:

/a(r)/

/a~(r)/

/i(r)/

/o(r)/

/u(r)/

Lists of phones, words and phrases

PHONES are incorporated in these words:

b**E**ad

b**I**d

b**E**d

b**A**rd

p**O**t

p**O**rt

p**U**t

b**OO**t

b**U**d

b**I**rd

b**AI**t

b**I**te

b**O**y

b**O**w

**Po**p

**B**ib

**T**eat

**D**ied

**C**ake

**G**o

**Ch**in

**J**udge

**F**ine

**V**ine

**TH**ink

**Th**en

**S**ee

**Z**oo

**Sh**y

**TR**easure

**M**y

**N**ear

si**ng**

**L**ow

**R**aw

**W**est

**Y**ear

**H**igh

40 WORDS:

Dale

Bought

Pun

Stock

Ton

Bit

Bore

Peach

Dill

Stole

Base

Bark

Scale

Skate

Scoop

Dew

Park

Got

Tart

Tare

Duff

Spill

Done

Steel

Pie

Tough

Core

Bout

Start

Die

Spur

Dub

Score

Ghoul

Pot

Stab

Damp

Deer

Gold

Stamp

PHRASES (that contain all phones)

HELLO WORLD

HOW ARE YOU DOING TODAY SIR

TRY TO MAKE MY CONCERT TONIGHT

I THINK SHE FINDS THE ZOO FUN

WHICH PRIVATE FIRM

THE JOY OF A JOG MAKES A BOY SAY “WOW”
